# Competency of peripheral health workers in detection & management of common syndromic conditions under surveillance, North 24 Parganas, West Bengal, India, 2016: a cross-sectional study

**DOI:** 10.1017/gheg.2017.13

**Published:** 2017-10-11

**Authors:** F. Debnath, T. Bhatnagar, L. Sundaramoorthy, M. Ponnaiah

**Affiliations:** 1 Division of Epidemiology, ICMR-National Institute of Cholera and Enteric Diseases, Kolkata, West Bengal, India; 2 Masters of Public Health Scholar, ICMR-National Institute of Epidemiology, Chennai, India; 3 ICMR School of Public Health, ICMR-National Institute of Epidemiology, Chennai, India

**Keywords:** Competency, peripheral health worker, syndromic surveillance, trainings

## Abstract

**Background:**

Competency of peripheral health workers in the detection and management of common syndromic conditions is crucial as they are the first point of contact for the majority of the Indian population.

**Methods:**

We measured the competency of auxiliary nurse midwives (ANMs), and factors associated with inadequate competency, in the detection and management of common conditions-diarrhoea, acute respiratory tract infection, fever, malaria-through a cross-sectional study using condition specific validated clinical vignettes and structured questionnaires.

**Results:**

Out of 272 selected ANMs, 68% (95% CI 62–74%) were adequately competent. Factors independently associated with inadequate competency were unavailability of essential drugs in preceding month [adjusted odds ratio (AOR) = 1.95; 95% CI 1.1–3.5] and ever trained in integrated management of childhood illness (AOR = 2.4; 95% CI 1.4–4.1).

**Conclusion:**

More than two third of the peripheral health workers were adequately competent to detect and manage common conditions. Ensuring uninterrupted drug availability and improved quality in service trainings might facilitate competency levels.

## Introduction

Globally, peripheral health workers are the final common pathway for implementation of all health programmes in the primary health care delivery system. While declaring 2006–2015 as the decade of health workers, World Health Organization (WHO) emphasized the importance of their performance [1]. WHO defined performance in four dimensions, viz., responsiveness, productivity, competence and availability. Competence accounts for knowledge, skill, ability and is also used as a marker of performance in many settings [2, 3] and affects the desirable outcome of any health programme [2]. Among the peripheral health workers, nurses play a central role in delivering services at all levels in the health care delivery system [4]. In view of the fact that pneumonia and diarrhoea are two major killers of children under-five worldwide [5], the health workers’ competence becomes critical in the detection and management of common syndromic conditions such as diarrhoea, acute respiratory tract infections (ARI), fever and malaria.

India is currently experiencing a severe shortage of human resources in the health care setting [6]. The existing health care workforce is characterized by a variety of health workers working at different levels [7]. The peripheral female health workers known as auxiliary nurse and midwives (ANMs) constitute 30% of all health workers [8] and the estimated density is 2.4 per 10 000 population [8]. They work at the health sub centre (HSC) level and perform several activities ranging from maternal and child health care to detection, management and reporting of common syndromic conditions like diarrhoea, cough less than 3 weeks, fever and malaria, under different national programmes including surveillance. Diarrhoea and ARI are the major killers of Indian children in the under-5 age-group [9]. Hence, the workers’ competence in the detection and management of those conditions is as critical as primary care givers when the nation's health care work force is experiencing severe shortages. However, we currently do not have any published information on the competence of peripheral health workers in the detection and management of common syndromic conditions.

West Bengal province in Eastern India has a structured health care delivery system. Peripheral female health workers [known as first ANMs (1st ANMs)] work at the HSC level and report to the authorities at the sub-district (called ‘block’) level. Block health authorities report to the District Health Officials and in turn, the District reports to the State. An organizational review of the Health Department of the Government, carried out in 2006, found 1st ANMs spent 35% of their time preparing reports and meetings [10]. The review also identified issues at the HSC level, related to the physical conditions, work load, supervision and monitoring; however, they did not quantify the impact of these issues on performance level.

In North 24 Parganas District of West Bengal, our evaluation of acute diarrhoeal surveillance under integrated disease surveillance programme (IDSP) revealed that only one fifth of the 1st ANMs knew the correct definition of diarrhoea [11]. According to district surveillance data for 2014, of all the syndromic conditions reported at HSCs, fever less than a week, cough with fever less than a week and diarrhoea constituted 42%, 36% and 20%, respectively [12]; however, no information was available on the accuracy of the detection of these conditions.

When the health system is experiencing a severe shortage of human resources, having competent peripheral health workers becomes critical [6]. In this context, we measured the level of competence among the 1st ANMs to detect and manage cases of diarrhoea, ARI, fever and malaria. As a secondary objective, we identified factors associated with 1st ANMs inadequate competency in detection and management of these syndromic conditions.

## Methods

### Study design

We conducted a cross-sectional study in North 24 Parganas District of West Bengal province of India.

### Sampling procedure

We used simple random sampling from a sampling frame of 738 1st ANMs. We generated a random numbers list using Open Epi Version 3 software [13].

### Sample size

We assumed 40% of the 738 1st ANMs in the study district to have adequate competency, based on results of a similar study done in Bangladesh on community health workers [14] and based on that, we needed to recruit 272 1st ANMs accounting 5% absolute precision, 95% confidence interval (CI) and 10% non-response. We used Open Epi Version 3 for calculation [13].

### Operational definitions

#### Diarrhoea

We defined diarrhoea as passage of three or more loose or watery stools in the past 24 h with or without dehydration [15].

#### Acute respiratory tract infection

We defined a case of ARI as a person with sudden onset of fever of >38 °C and cough or sore throat in the absence of other diagnosis [16].

#### Suspect malaria

We defined it as any patient with fever, with no other obvious cause, to be considered a case of suspected malaria. Any community health worker observing a case of suspected malaria must initiate a diagnostic test, either through the microscopy of blood to identify malarial parasites and/or a rapid diagnostic test [17].

#### Competency

We defined competency based on their response in a clinical vignette [3, 18–21].

#### Overall competence score (%)

We defined over all competence score (%) by combining marks obtained through the clinical vignettes for each of the four conditions of interest: diarrhoea, ARI, fever and malaria.

#### Adequate competency

Overall score of ⩾75% [19].

#### Inadequate competency

Overall score of <75% [19].

### Data collection procedure

#### Measuring competence

We measured competence using clinical vignettes specific to the studied conditions [3, 18–21]. Four major steps were followed while developing the vignettes: identification of conditions of interest, development of sections, constructing vignettes and validation [21].

##### Clinical vignettes

We developed the four-condition specific clinical vignettes after reviewing published literature. The vignettes contained sections on history taking, eliciting signs and management [21] and options were provided for each question in the vignettes. While collecting data, all the study participants were given all four clinical vignettes to complete.

##### Determination of factors associated with inadequate competence

We collected information on socio-demographics, work and health system related factors through interviews using a structured questionnaire and data abstraction form.

We collected information on health system level factors such as availability of drugs for the month preceding the survey, number of supervisory visits during September–November 2015, participation in integrated management of neonatal and childhood illness (IMNCI) training. We also abstracted information on training on IMNCI from administrative records of the district.

### Quality assurance

Prior to the initiation of the study, the study protocol underwent peer review. Clinical vignettes were adequately validated and pilot tested before administration. Double data-entry was employed when uploading data into the software and data were cleaned where necessary.

### Data analysis

#### For measuring competence level

We calculated descriptive statistics for all the continuous variables. We calculated competence scores based on identified correct answers in the vignettes. We awarded one mark for the section focused on the personal details of the patient, 2 marks for history taking as well as advice on home care/prevention, 2.5 marks for eliciting signs and 3 marks for treatment/management. For questions with multiple answers, 1 mark was deducted from the total for each wrong answer. Finally, marks on eliciting personal details and history related answers were combined to calculate the total mark for the history taking section. Marks obtained on treatment/management and advice on homecare/preventive aspect were combined and to obtain a total score for the management section. The total score of each vignette was converted in to percentage. Competence score (%) was calculated for each syndromic condition and an overall competence score (%) was calculated by combining marks obtained in all four vignettes. We described overall competence score (%), competence score for each condition (%), competence score (%) in each section in quartiles. We calculated 95% CI for the proportions. We drew boxplots for each of the syndromic conditions of interest.

#### To determine the factors associated with inadequate competence

The questionnaire had eight parameters which investigated the satisfaction level of study participants regarding annual leave, salary, recognition, time spent with family, workplace security, application of knowledge and skill, reporting system, getting respect from community they work for. For each parameter participants were asked a set of questions to explore their satisfaction level, measured using a five-point Likert scale. A score of four or five was considered an agreement with the construct and the variables were categorized accordingly. We used simple logistic regression to compute crude odds ratio (OR) with 95% CI. We examined multi-collinearity among the variables and used multiple logistic regression to calculate the adjusted OR (AOR) with 95% CI for each of the variables found significant in the univariate analysis, adjusted for potential confounders. We used interaction term to account for effect modifiers. As, this was our secondary objective, among the above mentioned eight parameters for which satisfaction level was investigated, finally we reported only those which had an association with overall inadequate competency.

We used Epi Info 7 and SPSS.22 (Student Version) for analysis.

### Bias

We anticipated selection bias; hence, we created the sampling frame of 1st ANMs and used simple random sampling to select study participants. We anticipated errors in the measurement of competency while using clinical vignette and not actual performance and therefore expected misclassification of outcomes (competency level). In the absence of availability of standard clinical vignettes for measuring competency, we developed a tool based on published work from relevant settings [21]. We developed them in English and then translated to Bengali. We ascertained the content validity by a review of the clinical vignette by District public health officials (21). Further, we pilot-tested the clinical vignettes in two-areas. We made necessary corrections based on the pilot-testing and set 45 min as the maximum time given for answering all vignettes. We used cut off of scores used in published studies [16] in similar settings to categorize the participants in to adequately and inadequately competent groups. We measured all exposures including anticipated confounders. The principal investigator (FD) interviewed all the participants to reduce interviewer bias. Further, to reduce recall bias, for certain exposures we used record-based information instead of relying on interviews. For instance, we abstracted information on training on IMNCI from administrative records of the district. While analyzing data, we identified confounders and adjusted accordingly.

### Human participant's protection

We obtained approval from the Institutional Human Ethics Committee of the National Institute of Epidemiology, Chennai, India. We obtained approvals from the district administration to conduct the study. We obtained written informed consent from study participants before data collection. We maintained confidentiality.

## Results

### Baseline characteristics of the study participants

We recruited 272 study participants with a mean age (standard deviation) of 43.7 (9.8) years (range: 22–59). Little over 50% were aged between 46 and 59 years (Table 1). The majority of participants were married and less than 50% had a graduate degree or above. Of the 272, 14 (5%) stayed within the health sub-centre area. Most had more than 11 years (median = 22 years; inter-quartile range (IQR) = 10–28 years) of service experience. The median time of daily travel was 2 h (IQR: 1.1–4 h) with more than 50% of participants travelling every day for 2 h or more. The majority of study participants (82%) reported that their HSC served more people than the target population of 5000, set by the Indian Public Health Standards Guidelines for Sub-Centres [22] (Table 1).

**Table 1.** Baseline characteristics of study participants (N = 272): Cross-sectional study of competency among peripheral health workers, North 24 Parganas District, West Bengal, India, 2016

Characteristics	*n*	%
Age (years)		
22–30	40	15
31–45	90	33
46–59	142	52
Married	253	93
Attained graduate degree or more	115	42
Residing within Health sub centre area	14	5
Residing within Block area she works	103	38
Service experience (years)		
<5	17	6
5–10	58	21
>10	197	72
Daily travel time of ≥2 h	173	64
Cater >5000 population	222	82

### Competence level among study participants

**Fig. 1.**
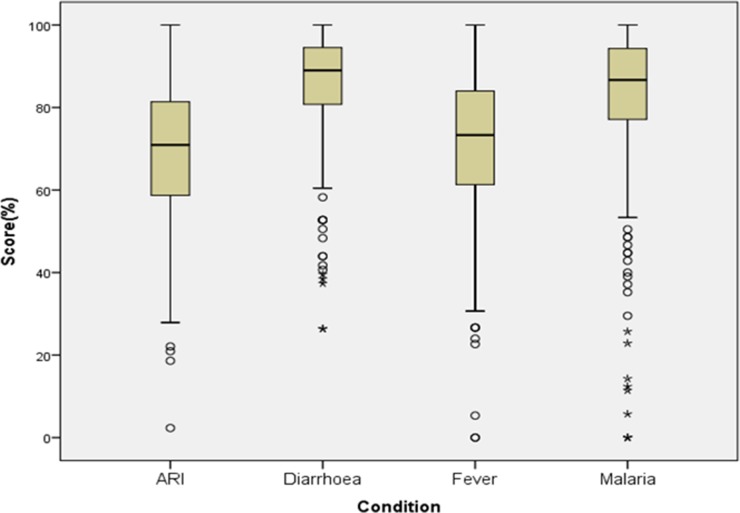
Median competence score (%) of 1st auxiliary nurse midwives in detection & management of common syndromic conditions – diarrhoea, ARI, fever and malaria: Cross-sectional study of competency among peripheral health workers, North 24 Parganas District, West Bengal, India, 2016. *, represents extreme outliers; o, represents mild outliers; ARI, acute respiratory tract infection.

In total 185 study participants (68%, 185/272) scored 75% or above (95% CI 62–74) in the overall competence score (Table 2). One quarter (*n* = 69) of the study participants scored between 50 and <75% (95% CI 20–31) and less than 1% of participants scored <25%. The competency score for detection and management was highest for diarrhoea (81%) and least for ARI (43%) (Table 2; Fig. 1). Further, when we looked in to the individual sections for all four conditions, we found only 60% of participants scored 75% or above in eliciting signs (Table 2). Within the domain of detection and management, in fever and malaria vignette, 179 (66%,179/272) and 249 (92%, 249/272) study participants scored 100% in eliciting sign section respectively, while the rest scored zero (results not shown in Table 2).

**Table 2.** Overall, condition specific, section specific competence score (%) of 272 1st Auxiliary Nurse Midwives: Cross-sectional study of competency among peripheral health workers, North 24 Parganas District, West Bengal, India, 2016

Score category	Overall competence score			Competence score by condition								Competence score by sections					
				Diarrhoea		ARI		Fever		Malaria		History taking		Eliciting sign		Management	
	*n*	%	95% CI	*n*	%	*n*	%	*n*	%	*n*	%	*n*	%	*n*	%	*n*	%
<25%	2	0.7	0.1–3%	0	0	4	1	5	2	9	3	5	2	5	2	4	2
25–49.9%	16	6	3–9%	10	4	38	14	34	13	12	4	21	8	25	9	12	4
50–74.9%	69	25	20–31%	40	15	113	42	108	40	43	16	62	23	80	29	63	23
≥75%	185	68	62–74%	222	82	117	43	125	46	208	77	184	68	162	60	193	71

CI, confidence interval; ARI, acute respiratory tract infection.

### Factors associated with overall inadequate competency (overall competence score <75%)

Age above median (OR = 2.7; 95% CI 1.5–4.9), service experience of 22 years or more (OR = 1.8; 95% CI 1.1–3.1), ever trained in integrated management of neonatal and childhood (IMNCI) (OR = 2.4; 95% CI 1.4–3.9), experienced a stock out of two to three drugs in the preceding month (OR = 1.9; 95% CI 1.1–3.4), serving a population more than 5000 (OR = 3.5; 95% CI 1.5–8.7), lacking the support of the block health authority (OR = 1.8; 95% CI 1.1–3.1), dissatisfied with the amount of annual leave (OR = 2.1; 95% CI 1.1–4.1) were all associated with overall inadequate competency (Table 3).

**Table 3.** Factors associated with inadequate competency: Cross-sectional study of competency among peripheral health workers, North 24 Parganas District, West Bengal, India, 2016

Characteristics	Outcome				Crude OR [95% CI] {*p* value}	Adjusted OR [95% CI] {*p* value}
	Inadequately competent (*N* = 87)		Adequately competent (*N* = 185)			
	*n*	%	*n*	%		
Sociodemographic characteristics						
Age (years) ≥43.7	68	78.2	105	56.8	2.7 [1.5–4.9] {<0.01}	–
Service experience (years) ≥22	53	60.9	85	46	1.8 [1.1–3.1] {0.02}	–
Health system level factors						
Received IMNCI training ever (Yes)	45	51.7	58	31.3	2.4 [1.4–3.9] {<0.01}	2.4 [1.4–4.1]^a^ {<0.01}
No. of stock out drug in preceding month of interview						
≤1	23	26.4	69	37.3	Ref	Ref
2 to 3	54	62.1	84	45.4	1.9 [1.1–3.4] {0.02}	1.9 [1.1–3.5]^b^ {0.02}
>3	10	11.5	32	17.3	0.9 [0.4–2.2] {0.88}	0.9 [0.4–2.1]^b^ {0.88}
Population served by ANM (>5000)	80	91.9	142	76.8	3.5 [1.5–8.7] {<0.01}	–
Block supports ANM's work (No)	47	54.0	72	38.9	1.8 [1.1–3.1] {0.02}	–
Individual level factors						
ANM feels satisfied with annual leave she gets (dissatisfied)	73	83.9	132	71.3	2.1[1.1- 4.1] {0.02}	–

CI, confidence interval; OR, odds ratio; IMNCI, integrated management of neonatal and childhood illnesses; ANM, auxiliary nurse midwife; Ref, reference category.

a Adjusted for service experience.

b Adjusted for block supporting 1st ANM's work.

After adjusting for potential confounders, we found those who received training in IMNCI were two times more likely to be inadequately competent (AOR = 2.4; 95% CI 1.4–4.1). Those who experienced a stock out of two to three drugs in the preceding month of interview compared with those who did not experience any drug stock out or stock out of only one drug (reference category) were almost two times more likely to be inadequately competent (AOR = 1.9; 95% CI 1.1–3.5) (Table 3).

We did not identify any effect modifier for the association of inadequate competence and different exposure variables.

## Discussion

In a cross-sectional study, using clinical vignettes, we measured the competency level and factors associated with inadequate competency among 1st ANMs for the detection and management of a few important syndromic conditions in North 24 Parganas District of West Bengal. We estimated that two-thirds of 1st ANMs were adequately competent to detect and manage all four conditions. Less than half of the participants were adequately competent in the detection and management of ARI and fever. However, in the individual sections of the competency tool, we identified that study participants scored lowest in eliciting signs compared with history taking and management. Receiving training in IMNCI and experiencing stock out in the preceding month were independently associated with overall inadequate competency.

Overall, more study participants than assumed were adequately competent in detection and management of all four conditions. But when we looked in to each condition, we found less number of them scored adequately in detection and management of ARI and fever. This could be attributed to the fact that the majority of the 1st ANMs were unaware or forgot age-specific respiratory rate. Moreover, most of them were confused about confirming malaria by blood test in fever cases. They actually did not know that all fever cases should be suspected for malaria, after ruling out other common causes and confirmed through blood test as per the guidelines of National Vector-borne Disease Control Programme. Compared with history taking and management section, fewer study participants scored adequately in eliciting signs section. This may be partially explained by their work load. Most of the study participants serve population exceeding the set norm [22, 23]. Those who serve larger population, tend to have greater workloads to complete within their fixed working hours. It is therefore possible that their quality of work can deteriorate over time, which in turn, can further affect their competency [24]. Unexpectedly, we also found that ANMs with more service experience were more likely to be inadequately competent. Moreover, in our study we found that those who reported lack of support from the block health authority had greater odds of being classified as inadequately competent. Regular supervision and support from supervisors have been globally identified as important factors for good performance among health care workers at all levels [19, 24]. Systematic supervision using clearly defined quantifiable indicators can actually improve competence of health workers [19, 24]. However, if the supervision mechanism is well structured, it is more likely to confer support to the health workers in the form of regular feedback, which may have an impact on individual competency. The matter of concern is, consequences of not knowing the age-specific respiratory rates/what to be done in fever case detection and management can be serious. That can ultimately lead them to use antibiotics inappropriately, specifically amoxicillin/cotrimoxazole, whenever children present with cough and fever [19].

Study participants who received training in IMNCI, scored inadequately. As per the Government of India's National Health Mission programme, it was mandatory for health staff at various levels (including peripheral health workers) to undergo training on IMNCI [25] to acquire knowledge and skill for proper detection and management of illness among new born and children. Moreover, IMNCI is an ongoing programme in most of the districts of West Bengal [26]. Given such importance and widespread popularity of this programme, we presume that the quality of such trainings needs to be reviewed. Though the training was done in different batches, none of them included a pre-test or post-test. Moreover, in our study we also found that fewer participants scored adequately in detection and management of ARI, fever. There are studies in India which also questioned quality of training provided to auxiliary nurses and midwives under different programmes [21] as training opportunities are one of the indicators for acquiring and maintaining competence of human resources to meet the health and public health needs of the country [6]. Moreover, maintaining a competent frontline health work force becomes more important when the country's health system has a severe shortage of human resources [6].

Availability of drugs is another critical aspect for optimal functioning of health workers [19]. In this study, the majority study participants experienced a stock out of two to three drugs in the month preceding the interview. These types of drug stock outs were associated with inadequate competency. Studies in African countries showed similar results where lengthy drug stock out period and the erratic supply of drugs affected the performance of the health workers [18, 19].

Our study had few limitations. Firstly, the clinical vignettes used in the study may not be considered comprehensive for assessment of staff competency in detection and management of diarrhoea, ARI, fever and malaria as these were locally developed tool. As mentioned earlier we tried to reduce misclassification error. However, it is still possible that we might have underestimated the proportion of adequately competent 1st ANMs yet even in that scenario, nearly 70% of them having adequate competency is satisfactory. Moreover, these vignettes can be administered quickly in resource poor settings and they can hold patient level variations constant [27, 28]. Secondly, our study might suffer from information bias. We reduced both interviewer bias and recall bias by using a single interviewer and collecting key information from records than relying on interviews alone respectively. However, we believe that our finding on association of IMNCI training with competency may still have to be verified through performance review by local authorities [29].

We conducted this study following epidemiological methods. Hence, our findings are entirely applicable to peripheral health workers in the study District and may be relevant to the State. In fact, the results of this study were similar to many such studies, conducted either in India or similar settings [6, 8, 10, 14, 18–23].

We found peripheral female health workers were adequately competent in history taking and the detection and management of diarrhoea; however, improvements to 1st ANMs’ competency in detecting and managing ARI and fever are required. Receiving training in IMNCI and experiencing two to three drugs stock out in the preceding month of interview attributed to overall inadequate competency.

On the basis of our findings, we recommend maintaining and improving adequate competency of health workers through multiple methods such as refresher training, supportive supervision and ensuring support systems in the form of uninterrupted supply of drugs and periodic revisit of population norms and appropriate allocation of workers. As a long-term measure, we also recommend evaluating quality of training programmes for the health workers. Additionally, we recommend in-depth studies on their behaviour and interventions to improve individual level competency, especially due to continued shortage of health workers in the health system.

## Supplementary material

For supplementary material accompanying this paper visit http://dx.doi.org/10.1017/gheg.2017.13. click here to view supplementary material

## References

[1] The World Health Report 2006. World Health Organization. 2006, vol. 19, p. 237 (http://www.who.int/whr/2006/whr06_en.pdf). Last accessed 1 July 2016.

[2] Kak N, Burkhalter B, Cooper M. Measuring the competence of healthcare providers. Quality Assurance 2001; 2: 1–28.

[3] Krishna DR, Which doctor for primary health care? Quality of care and non-physician clinicians in India. Social Science and Medicine 2013; 84: 30–34. (http://dx.doi.org/10.1016/j.socscimed.2013.02.018). 23517701 10.1016/j.socscimed.2013.02.018

[4] Strengthening the WHO nursing and midwifery agenda. World Health Organization 2015. (http://www.who.int/hrh/news/2015/midwifery_nurse_agenda/en/). Last accessed 1 July 2016.

[5] Children : reducing mortality. World Health Organization Fact sheet 2016. (http://www.who.int/mediacentre/factsheets/fs178/en/). Last accessed 1 July 2016.

[6] Rao M, Human resources for health in India. The Lancet 2011; 377(9765): 587–598. doi: 10.1016/S0140-6736(10)61888-0. 21227499

[7] Introduction To National Classification of Occupations, 2004, pp. 1–22. (http://www.dget.nic.in/upload/uploadfiles/files/publication/1%20preface.pdf). Last accessed 1 July 2016.

[8] Krishna DR, Which doctor for primary health care ? An assessment of primary health care providers in Chhattisgarh, India. June 2010. Available from: http://health.cg.gov.in/ehealth/studyreports/Which%20Doctor%20For%20Primary%20Health%20Care.pdf

[9] Ahmed SF, Prevalence of diarrhoeal disease, its seasonal and age variation in under- fives in Kashmir, India. International Journal of Health Sciences *(*Qassim*)* 2008; 2: 126–133. PMC3068726 21475494

[10] Ferguson AF, Co. Draft Report. Organizational/Institutional Review of the Department of Health and Family Welfare, Government of West Bengal: Organization Development and Human Resource, 2006.

[11] Debnath F, Ponnaiah M. Improved timeliness for reporting of acute diarrhoeal disease surveillance over time: Evaluation of Integrated Disease Surveillance Programme in North 24 Parganas, West Bengal, India, 2015, 14–19 p. Located at Library of National Institute of Epidemiology, Chennai, India.

[12] Office of the Chief Medical Officer of Health, North 24 Parganas District, West Bengal.

[13] Dean AG, Sullivan KM, Soe MM. V. OpenEpi: Open Source Epidemiologic Statistics for Public Health, Version. 2014 (http://www.openepi.com/Menu/OE_Menu.htm, www.OpenEpi.com), updated 2015/05/04, Accessed 19 May 2016.

[14] Alam B, Assessment of performance of community health workers of MANOSHI. MANOSHI working paper. No. 16, 2012. (http://citeseerx.ist.psu.edu/viewdoc/download?doi=10.1.1.701.4909&rep=rep1&type=pdf). Last accessed 5 May 2017.

[15] Case Definitions – P form. Integrated Disease Surveillance Programme. Ministry of Health & Family Welfare. Government of India. (http://www.idsp.nic.in/). Last accessed 23 May 2017.

[16] WHO recommended surveillance standards, 1999. (http://www.who.int/hiv/strategic/surveillance/pubstandards/en/). Last accessed 23 May 2017.

[17] Guidilines: National Vector borne disease control programme. (http://nvbdcp.gov.in/iec.html). Last accessed 23 May 2017.

[18] Traoré M, Obstetric competence among primary healthcare workers in Mali. International Journal of Gynecology & Obstetrics 2014; 126(1): 50–55. (http://linkinghub.elsevier.com/retrieve/pii/S0020729214001702). 24800658 10.1016/j.ijgo.2014.01.012

[19] Bagonza J, Kibira SPS, Rutebemberwa E. Performance of community health workers managing malaria, pneumonia and diarrhoea under the community case management programme in central Uganda: a cross sectional study. Malaria Journal 2014; 13(1): 367 (http://www.malariajournal.com/content/13/1/367). 25231247 10.1186/1475-2875-13-367 PMC4174662

[20] Das J, Hammer J. Location, location, location: residence, wealth, and the quality of medical care in Delhi, *India*. Health Affairs *(*Millwood*)* 2007; 26(3): w338–w351. (http://content.healthaffairs.org/cgi/doi/10.1377/hlthaff.26.3.w338). 10.1377/hlthaff.26.3.w338 17389631

[21] Chaturvedi S, Upadhyay S, De Costa A. Competence of birth attendants at providing emergency obstetric care under India's JSY conditional cash transfer program for institutional delivery: an assessment using case vignettes in Madhya Pradesh province. BMC Pregnancy and Childbirth 2014; 14(1): 174 (http://www.biomedcentral.com/1471-2393/14/174). 24885817 10.1186/1471-2393-14-174 PMC4075933

[22] Indian Public Health Standars Guidelines For Sub Centers, 2012. Directorate General of Health Services Ministry of Health & Family Welfare Government of India. (health.bih.nic.in/Docs/Guidelines/Guidelines-Sub-Centers-(Revised)-2012.pdf).

[23] Bm P, Muraleedharan V. Community Health Workers: a review of concepts, practice and policy concerns. 2007 (http://www.chwcentral.org/community-health-workers-review-concepts-practice-and-policy-concerns). Last accessed 1 July 2016.

[24] Bajpai N, Dholakia RH. Improving the performance of accredited social health activists in India. Working papers series, Columbia global centers, South Asia, Columbia university. Working Paper No. 1 May 2011. (http://globalcenters.columbia.edu/files/cgc/pictures/Improving_the_Performance_of_ASHAs_in_India_CGCSA_Working_Paper_1.pdf). Last accessed 5 May 2017.

[25] Approval for state programme implementation plan of west bengal:2012–13. (http://pipnrhm-mohfw.nic.in/PIP2012-13_files/ROP%202012-13/West%20Bengal/Approval%20Rop%20WB.pdf). Last accessed 22 May 2017.

[26] Programme Implementation plan 2011 -2012. (http://pipnrhm-mohfw.nic.in/index_files/non_high_focus_large/westbengal/Presentation/Presentation_PIP_11-12-for-NPCC-Meeting.pdf). Last accessed 22 May 2017.

[27] Peabody JW, Improving patient care measuring the quality of physician practice by using clinical vignettes : a prospective validation study. Annals of Internal Medicine 2004; 141(10): 771–780. doi: 10.7326/0003-4819-141-10-200411160-00008. 15545677

[28] Peabody JW, Using vignettes to compare the quality of clinical care variation in economically divergent countries. Health Services Research Journal 2004; 39(6 pt 2): 1951–1970. 10.1111/j.1475-6773.2004.00327.x PMC1361107 15544639

[29] Bhatnagar T, Seven years of the field epidemiology training programme (FETP) at Chennai, Tamil Nadu, India: an internal evaluation. Human Resources for Health 2012; 10: 36. doi: 10.1186/1478-4491-10-36. 23013473 PMC3505457

