# Supplementary material for: Competency of peripheral health workers in detection & management of common syndromic conditions under surveillance, North 24 Parganas, West Bengal, India, 2016: a cross-sectional study
Source: Glob Health Epidemiol Genom. 2017 Oct 11;2:e15. doi: 10.1017/gheg.2017.13 (PMC5870437; doi:10.1017/gheg.2017.13)
Supplement: Supplementary file 1 [file S2054420017000136sup001.docx]

**Figure 2: Section specific median competence score (%) of the 1^st^ ANMs in detection & management of common syndromic conditions - diarrhoea, ARI, fever & malaria: Cross-sectional study of competency among peripheral health workers, North 24 Parganas district, West Bengal, India, 2016**

| 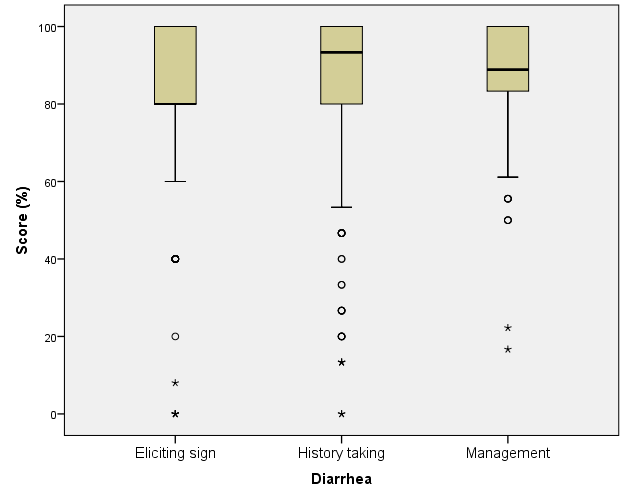 | 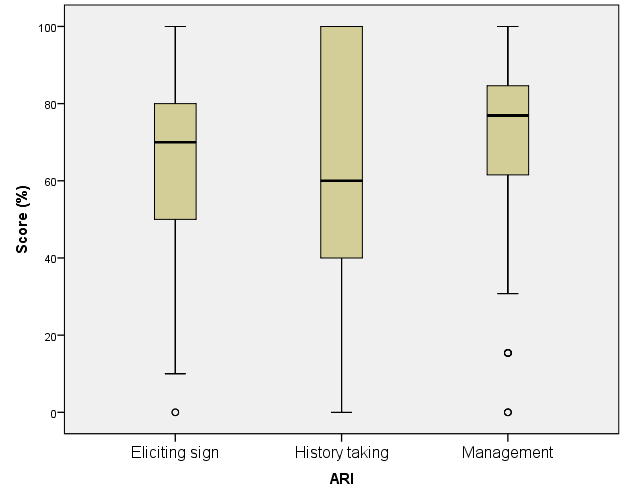 |
| --- | --- |
| Diarrhoea | ARI |
| 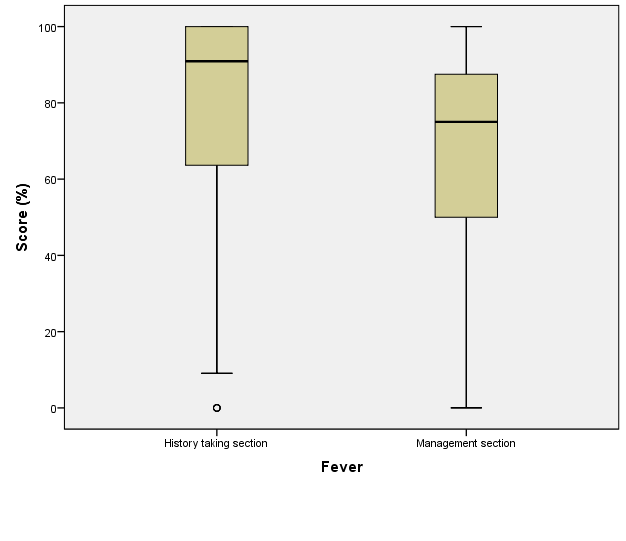 | 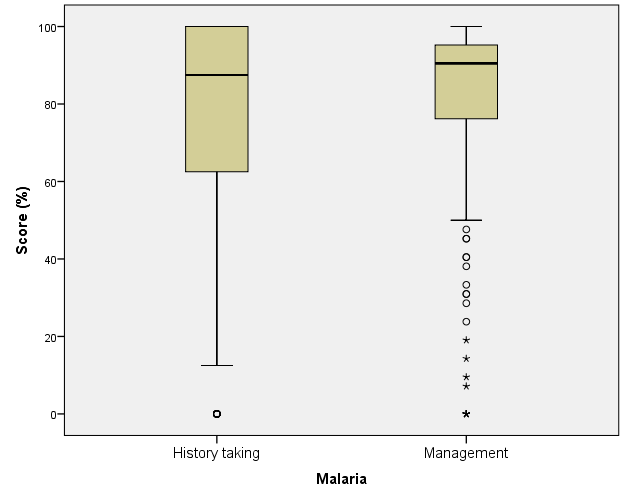 |
| Fever | Malaria |
